# Supplementary material for: Effects of Biased Analogues of the Kappa Opioid Receptor Agonist, U50,488, in Preclinical Models of Pain and Side Effects
Source: Molecules. 2025 Jan 29;30(3):604. doi: 10.3390/molecules30030604 (PMC11820436; doi:10.3390/molecules30030604)
Supplement: Supplementary file 1 [file molecules-30-00604-s001.zip › molecules-3403860-supplementary.pdf]

Supplementary Materials

Effects of biased analogues of the kappa opioid receptor agonist, U50,488, in preclinical models of pain and side effects

Ross van de Wetering [1], Loan Y. Vu [2], Lindsay D. Kornberger [2], Dan Luo [2], Brittany Scouller [1], Sheein Hong [1], Kelly Paton [1], Thomas E. Prisinzano [2], and Bronwyn M. Kivell [1,*].

[1] School of Biological Sciences, Centre for Biodiscovery, Victoria University of Wellington, Wellington, 6012, New Zealand.
[2] Department of Pharmaceutical Sciences, University of Kentucky, Lexington, KY 40506, USA.
* Correspondence: bronwyn.kivell@vuw.ac.nz

Contents:

1. Synthesis of **1**, **2**, and **3**.
2. HPLC and NMR of **1**, **2**, and **3**.
3. Table S1. Summary of statistical analyses.

1. Synthesis of 1, 2, and 3

General Experimental Procedures. Reactions were performed in oven-dried glassware under normal atmosphere (unless otherwise specified). All chemical reagents were purchased from commercial suppliers and used without further purification. Anhydrous solvents were either purchased from commercial suppliers or obtained from a solvent purification system in which solvent was passed through two columns of activated alumina under nitrogen. Reactions were monitored by thin-layer chromatography (TLC) on Supelco TLC Silica gel 60 F_{254} 20 × 20 cm silica gel plates (Sigma Aldrich) and visualized by UV (254 nm). Flash column chromatography was performed on a CombiFlash NextGen 300+ purification system (Teledyne Isco) using prepacked RediSepRf silica columns. ^{1}H and ^{13}C NMR were recorded on a Bruker 400 MHz spectrometer, 500 MHz Varian spectrometer, and/or Bruker 600 MHz spectrometer. Chemical shifts are reported in ppm and referenced with respect to the residual solvent: $CDCl_3$ at 7.26 ppm, MeOD at 3.31 ppm, DMSO-d6 at 2.50 ppm (1H NMR); $CDCl_3$ at 77.16 ppm, MeOD at 49.00 ppm, DMSO-d6 at 39.52 ppm (13C NMR). In other cases, $CDCl_3$ or DMSO-d6 containing tetramethylsilane (TMS) were utilized. Coupling constants (J) are reported in Hz. High-resolution mass spectra (HRMS) were obtained on an Agilent 6230 time-of-flight mass spectrometer with an electrospray ion source in positive mode. Compounds selected for biological testing were identified as >95% pure by high-performance liquid chromatography (HPLC) on an Agilent 1260 Infinity II with diode array detection at 214 nm. A Poroshell 120EC-C18 column (4.6 × 100 mm2, 2.7 mm) with a 5 min gradient mobile phase of 5−100% acetonitrile/0.1% trifluoroacetic acid (TFA) in water was utilized.

(*S*)-2-(3,4-Dichlorophenoxy)-*N*-methyl-*N*-(1-phenyl-2-(pyrrolidin-1-yl)ethyl)acetamide (1). HATU (188.3 mg, 0.495 mmol) was added to a solution of 3,4-dichlorophenoxyacetic acid (109.5 mg, 0.495 mmol) in

DMF (3 mL). The reaction mixture was stirred for 5 min and *i*-Pr_2NEt (0.173 mL, 0.991 mmol) was then added. After an additional 5 min of stirring, a solution of (*S*)-*N*-methyl-1-phenyl-2-(pyrrolidin-1-yl)ethan-1-amine (100.0 mg, 0.450 mmol) in DMF (2 mL) was added in a dropwise manner. The resulting solution was stirred at room temperature overnight. The reaction mixture was quenched with water (30 mL) and the resulting mixture was extracted with EtOAc (50 mL). The organic layer was washed with water (3 × 50 mL) then brine (30 mL). Removal of the solvent under reduced pressure afforded a crude product that was subjected to column chromatography utilizing an EtOAc/MeOH gradient to afford a yellow oil (44.6 mg, 24.3 % yield). HRMS cald (ESI) *m/z* for $[M + H]^+$ calcd $C_{21}H_{25}Cl_2N_2O_2$, 407.1288; found, 407.1319.

The resulting oil (44.6 mg, 0.107 mmol) was then converted to the oxalate salt: ^1H NMR (600 MHz, DMSO) δ 7.48 (d, *J* = 8.9 Hz, 1H), 7.43 – 7.32 (m, 4H), 7.29 – 7.22 (m, 2H), 7.03 (dd, *J* = 9.0, 3.0 Hz, 1H), 6.07 (dd, *J* = 12.2, 3.4 Hz, 1H), 5.12 – 5.03 (m, 2H), 4.05 (t, *J* = 12.7 Hz, 1H), 3.53 (dd, *J* = 13.4, 3.4 Hz, 1H), 3.40 (s, 2H), 3.25 (s, 2H), 2.70 (s, 3H), 2.00 – 1.89 (m, 4H). ^{13}C NMR (151 MHz, DMSO) δ 168.6, 164.7, 157.8, 136.3, 131.4, 130.5, 128.7, 128.1, 127.3, 122.3, 116.4, 116.0, 65.6, 53.6, 52.1, 51.4, 28.0, 22.7. HPLC purity: 98%, t_r = 3.485 min. $[a]_D^{25}$ = + 140.5 (c=0.0025, MeOH).

(S)-2-(2,6-Dichlorophenyl)-*N*-methyl-*N*-(1-phenyl-2-(pyrrolidin-1-yl)ethyl)acetamide (2). HATU (188.3 mg, 0.495 mmol) was added to a solution of 2,6-dichlorophenyl acetic acid (101.6 mg, 0.495 mmol) in DMF (3 mL). The reaction mixture was stirred for 5 min and *i*-Pr_2NEt (0.173 mL, 0.991 mmol) was added. After an additional 5 min of stirring, a solution of (*S*)-*N*-methyl-1-phenyl-2-(pyrrolidin-1-yl)ethan-1-amine (100.0 mg, 0.450 mmol) in DMF (2 mL) was added in a dropwise manner. The resulting solution was stirred at room temperature overnight. The reaction mixture was quenched with water (30 mL) and the resulting mixture was extracted with EtOAc (50 mL). The organic layer was washed with water (3 × 50 mL) then brine (30 mL). Removal of the solvent under reduced pressure afforded a crude product that was subjected to column chromatography utilizing an EtOAc/MeOH gradient to afford a yellow oil (58.0 mg, 32.9 % yield). HRMS cald (ESI) *m/z* for $[M + H]^+$ calcd $C_{21}H_{25}Cl_2N_2O$, 391.1338; found, 391.1365.

The resulting oil (35.5 mg, 0.091 mmol) was then converted to the oxalate salt: ^1H NMR (600 MHz, MeOD) δ 7.47 – 7.38 (m, 5H), 7.37 – 7.33 (m, 2H), 7.29 (t, *J* = 8.1 Hz, 1H), 6.27 (dd, *J* = 11.2, 3.7 Hz, 1H), 4.24 (d, *J* = 16.9 Hz, 1H), 4.17 (dd, *J* = 13.3, 11.2 Hz, 1H), 4.11 (d, *J* = 17.0 Hz, 1H), 3.79 (dd, *J* = 13.3, 3.7 Hz, 1H), 3.47 (d, *J* = 58.4 Hz, 4H), 2.92 (s, 3H), 2.11 (s, 4H). ^{13}C NMR (151 MHz, MeOD) δ 173.2, 166.5, 137.3, 136.7, 133.8, 130.4, 130.2, 129.9, 129.2, 128.7, 56.2, 54.9, 54.2, 37.6, 30.8, 24.0. HPLC purity: 98%, t_r = 3.313 min. $[a]_D^{25}$ = + 145.9 (c=0.0025, MeOH)

(±)-1-(Pyridin-3-yl)-2-(pyrrolidin-1-yl)ethan-1-ol. Pyrrolidine (0.273 mL, 3.302 mmol) was added to a solution of 3-(oxiran-2-yl)pyridine (200.0 mg, 1.651 mmol) in EtOH (5.0 mL). The reaction was then heated at reflux for 1 h, cooled to room temperature and evaporated to dryness under reduced pressure. The crude product was subjected to column chromatography utilizing EtOAc/MeOH gradient to afford a dark brown oil (180.0 mg, 56.7 % yield): ^1H NMR (400 MHz, DMSO) δ 8.53 (d, *J* = 2.2 Hz, 1H), 8.43 (dd, *J* = 4.8, 1.7 Hz, 1H), 7.73 (dt, *J* = 7.9, 2.0 Hz, 1H), 7.37 – 7.29 (m, 1H), 5.23 (s, 1H), 4.68 (t, *J* = 6.6 Hz, 1H), 2.69 – 2.56 (m, 2H), 2.53 (d, *J* = 2.3 Hz, 2H), 2.46 (dd, *J* = 6.6, 2.5 Hz, 2H), 1.71 – 1.57 (m, 4H). ^{13}C NMR (101 MHz, DMSO) δ 148.5, 148.4, 140.4, 134.2, 123.6, 69.8, 64.2, 54.5, 23.7. [LCMS calcd for $C_{11}H_{16}N_2O$, 193.1; found, 193.2]

(±)-*N*-Methyl-1-(pyridin-3-yl)-2-(pyrrolidin-1-yl)ethan-1-amine. A mixture of Et_3N (0.510 mL, 3.667 mmol), methanesulfonoyl chloride (0.236 mL, 3.056 mmol), and (±)-1-(pyridin-3-yl)-2-(pyrrolidin-1-

yl)ethan-1-ol (235.0 mg, 1.222 mmol) in Et_2O (12.0 mL) was stirred at 0 °C under an argon atmosphere. The reaction was allowed to warm to room temperature over 30 min. Additional Et_3N (0.340 mL, 2.445 mmol) was added and the mixture was cooled to 0 °C. A solution of methylamine (40 wt. % in H_2O, 2.5 mL, 30.557 mmol) was then added in a dropwise manner. The resulting solution was allowed to warm to room temperature and stirred overnight. The reaction mixture was quenched with water (30 mL) and extracted with Et_2O (3 × 50 mL). The crude product was used for the next step without further purification. [LCMS calcd for $C_{12}H_{19}N_3$, 206.2; found, 206.2]

(±)-2-(3,4-Dichlorophenyl)-N-methyl-N-[1-(pyridin-3-yl)-2-(pyrrolidin-1-yl)ethyl]acetamide (3). HATU (262.4 mg, 0.690 mmol) was added to a solution of (3,4-dichlorophenyl)acetic acid (128.6 mg, 0.627 mmol) in DMF (4 mL). The reaction mixture was stirred for 5 min and *i*-Pr_2NEt (0.240 mL, 1.380 mmol) was then added. After an additional 5 min of stirring, a solution of (±)-N-methyl-1-(pyridin-3-yl)-2-(pyrrolidin-1-yl)ethan-1-amine (140.0 mg, 0.627 mmol) in DMF (3 mL) was added in a dropwise manner. The resulting solution was stirred at room temperature overnight. The reaction mixture was quenched with water (30 mL) and the resulting mixture was extracted with Et_2O (2 × 50 mL). The organic layer was washed with water (3 × 50 mL) then brine (30 mL). Removal of the solvent under reduced pressure afforded a crude product that was subjected to column chromatography utilizing a hex/EtOAc gradient to afford a yellow oil (100.0 mg, 40.6 % yield). HRMS cald (ESI) *m/z* for $[M + H]^+$ calcd $C_{20}H_{24}Cl_2N_3O$, 392.1291; found, 392.1303.

The resulting oil (22.9 mg, 0.058 mmol) was then converted to the oxalate salt: ^1H NMR (600 MHz, MeOD) δ 8.58 – 8.53 (m, 2H), 7.86 (dt, *J* = 8.0, 2.0 Hz, 1H), 7.52 (dd, *J* = 8.0, 4.9 Hz, 1H), 7.40 (d, *J* = 8.0 Hz, 2H), 7.30 – 7.24 (m, 1H), 6.36 – 6.30 (m, 1H), 4.29 – 4.22 (m, 2H), 4.09 (d, *J* = 17.0 Hz, 1H), 3.79 (dd, *J* = 13.4, 3.6 Hz, 1H), 3.53 (s, 3H), 3.35 (s, 1H), 3.00 (s, 3H), 2.11 (m, 4H). ^{13}C NMR (151 MHz, MeOD) δ 173.2, 166.6, 150.1, 149.5, 137.5, 137.3 (2C), 133.9, 133.8, 130.3, 129.2 (2C), 125.5, 56.0, 54.1, 52.7, 37.5, 31.3, 24.0. HPLC purity: 98%, t_r = 2.349 min.

General procedure for the Formation of Oxalate Salt. Free base compounds were dissolved in DCM or DCM/MeOH, followed by the addition of oxalic acid (1 equiv.) dissolved in MeOH. After stirring for 30 minutes, solvents were removed *in vacuo* and the resulting oily residue was triturated with diethyl ether with sonication at which point a precipitate formed. The mixture was concentrated *in vacuo*, and the resulting solid was suspended in additional diethyl ether and sonicated. Diethyl ether was removed *in vacuo* to afford the corresponding oxalate salt.

2. HPLC and NMR of 1, 2, and 3

HPLC Method
pump: Agilent 1260 Infinity II
column: Poroshell 120 EC-C18, 4.6 x 100 mm, 2.7 µm
sample size: 0.5 µL
sample concentration: 1 mg/mL
mobile phase: 5-100% acetonitrile / 0.1% TFA in water
flow rate: 1.5 mL/min
retention time: 3.485 min
purity: 98%

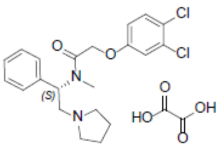

1

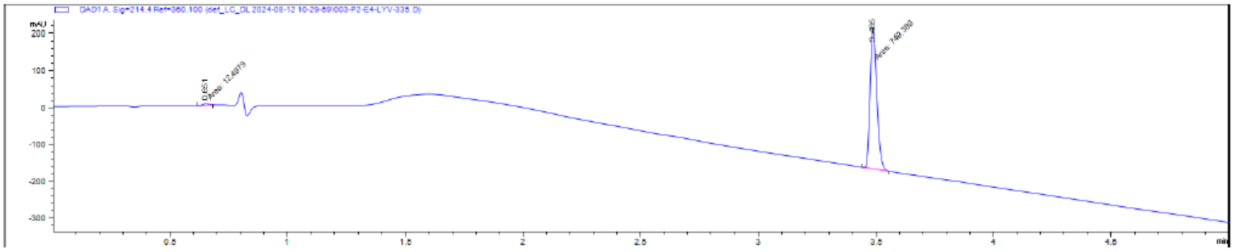

HPLC Method
pump: Agilent 1260 Infinity II
column: Poroshell 120 EC-C18, 4.6 x 100 mm, 2.7 µm
sample size: 0.5 µL
sample concentration: 1 mg/mL
mobile phase: 5-100% acetonitrile / 0.1% TFA in water
flow rate: 1.5 mL/min
retention time: 3.313 min
purity: 98%

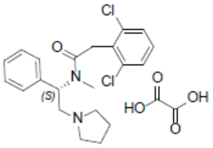

2

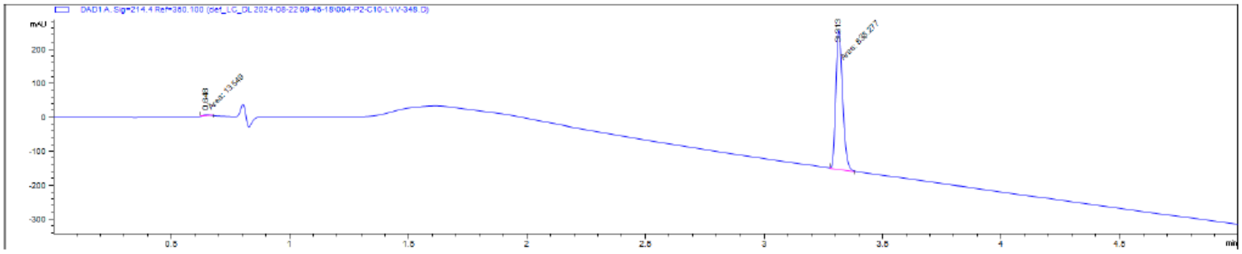

HPLC Method
pump: Agilent 1260 Infinity II
column: Poroshell 120 EC-C18, 4.6 x 100 mm, 2.7 µm
sample size: 1 µL
sample concentration: 0.5 mg/mL
mobile phase: 5-100% acetonitrile / 0.1% TFA in water
flow rate: 1.5 mL/min
retention time: 2.349 min
purity: 98%

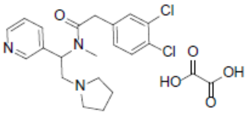

3

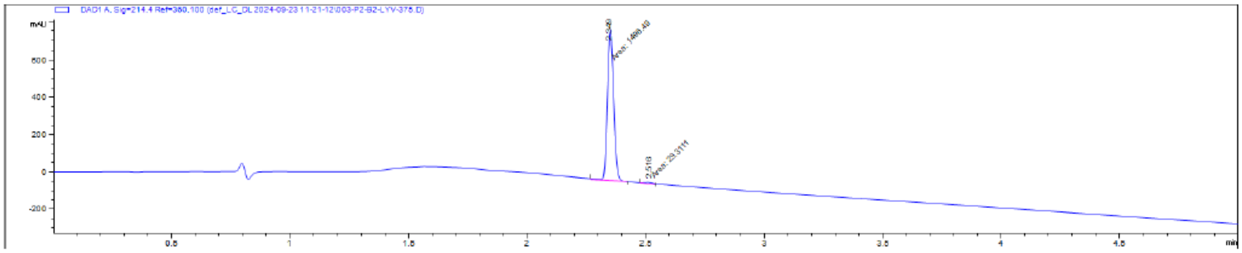

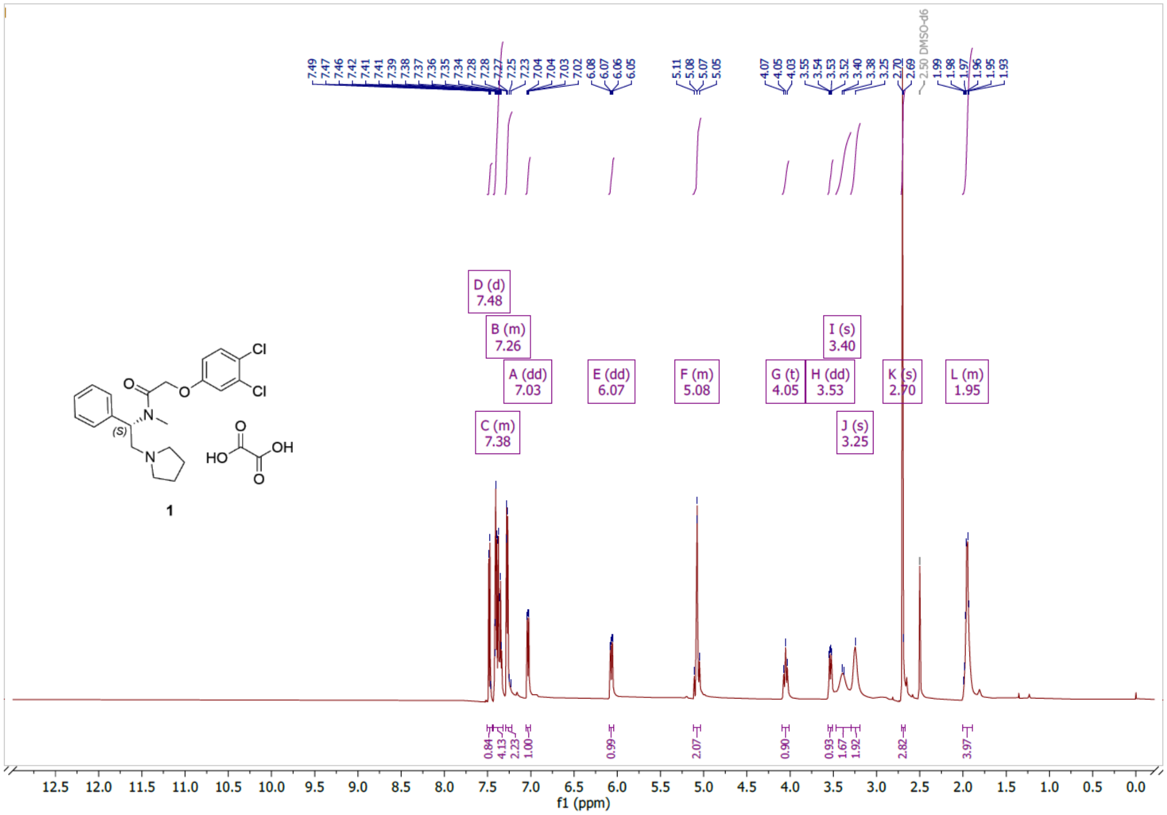
7.49 7.47 7.46 7.42 7.41 7.41 7.39 7.38 7.37 7.36 7.35 7.34 7.28 7.28 7.27 7.25 7.23 7.04 7.04 7.03 7.02 6.08 6.07 6.06 6.05 5.11 5.08 5.07 5.05 4.07 4.05 4.03 3.55 3.54 3.53 3.52 3.40 3.38 3.25 2.70 2.69 2.50 DMSO-d6 1.99 1.98 1.97 1.96 1.95 1.93
D (d) 7.48
B (m) 7.26
A (dd) 7.03
C (m) 7.38
E (dd) 6.07
F (m) 5.08
G (t) 4.05
H (dd) 3.53
I (s) 3.40
J (s) 3.25
K (s) 2.70
L (m) 1.95
Cl
Cl
O
O
N
(S)
N
HO
OH
O
O
1
0.84 4.13 2.23 1.00 0.99 2.07 0.90 0.93 1.67 1.92 2.82 3.97
12.5 12.0 11.5 11.0 10.5 10.0 9.5 9.0 8.5 8.0 7.5 7.0 6.5 6.0 5.5 5.0 4.5 4.0 3.5 3.0 2.5 2.0 1.5 1.0 0.5 0.0
f1 (ppm)

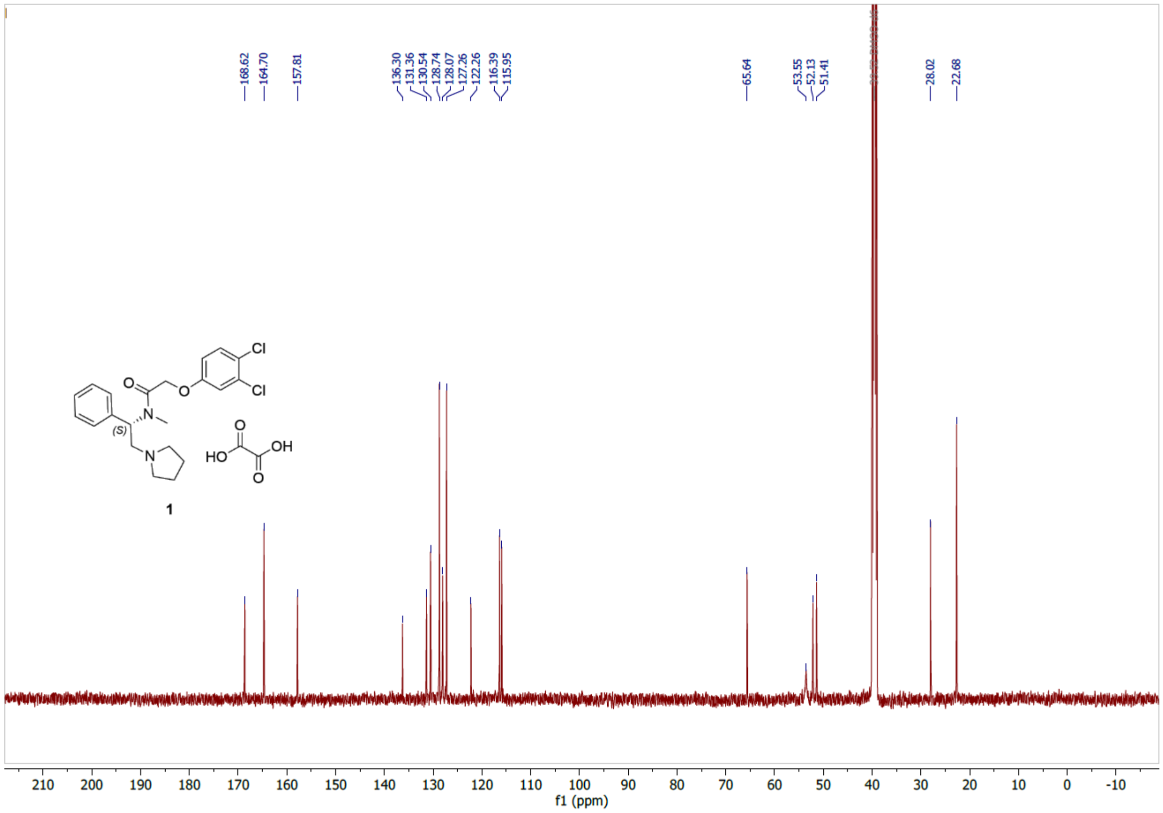
168.62 164.70 157.81 136.30 131.36 130.54 128.74 128.07 127.26 122.26 116.39 115.95 65.64 53.55 52.13 51.41 28.02 22.68
Cl
Cl
O
O
N
(S)
N
HO
OH
O
O
1
210 200 190 180 170 160 150 140 130 120 110 100 90 80 70 60 50 40 30 20 10 0 -10
f1 (ppm)

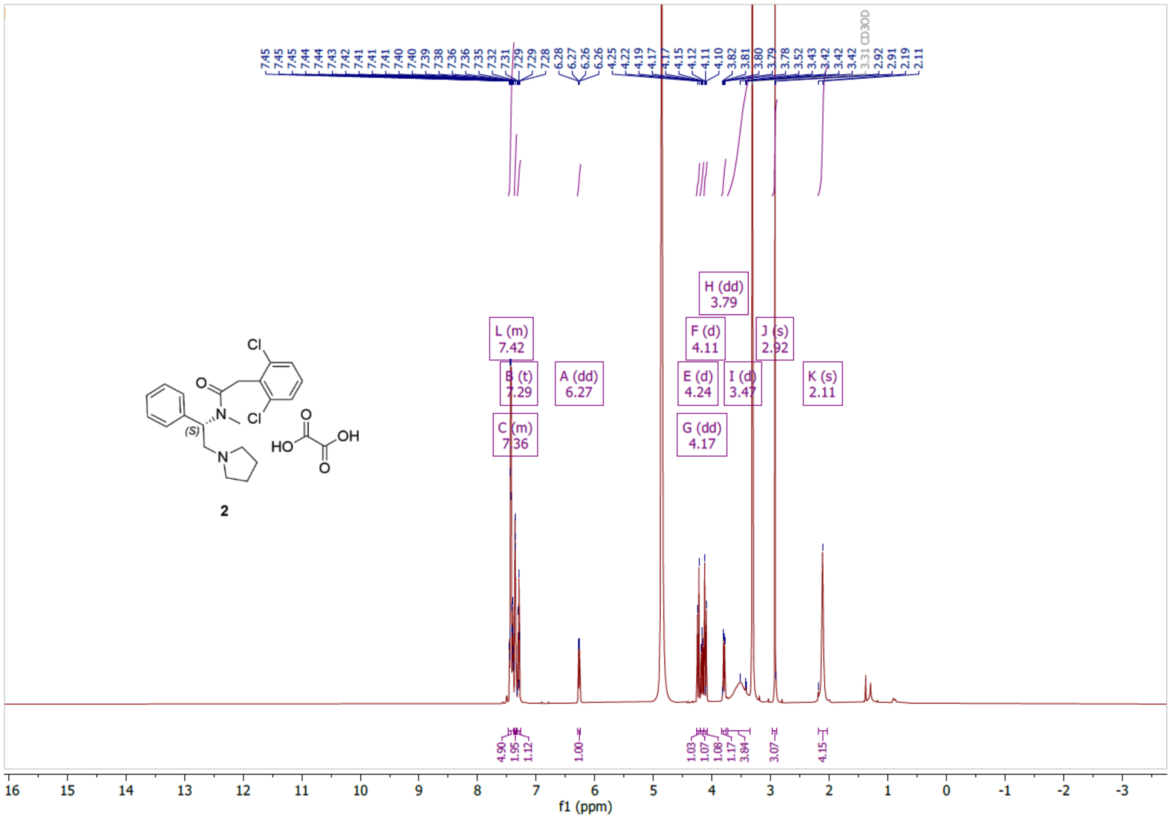
7.45
7.45
7.45
7.44
7.44
7.43
7.42
7.41
7.41
7.41
7.40
7.40
7.39
7.38
7.36
7.36
7.35
7.32
7.31
7.29
7.29
7.28
6.28
6.27
6.26
6.26
4.25
4.22
4.19
4.17
4.17
4.15
4.12
4.11
4.10
3.82
3.81
3.80
3.79
3.78
3.52
3.43
3.42
3.42
3.42
3.31 CD3OD
2.92
2.91
2.19
2.11
L (m) 7.42
B (t) 7.29
C (m) 7.36
A (dd) 6.27
H (dd) 3.79
F (d) 4.11
E (d) 4.24
G (dd) 4.17
I (d) 3.47
J (s) 2.92
K (s) 2.11
4.90
1.95
1.12
1.00
1.03
1.07
1.08
1.17
3.84
3.07
4.15
f1 (ppm)
2

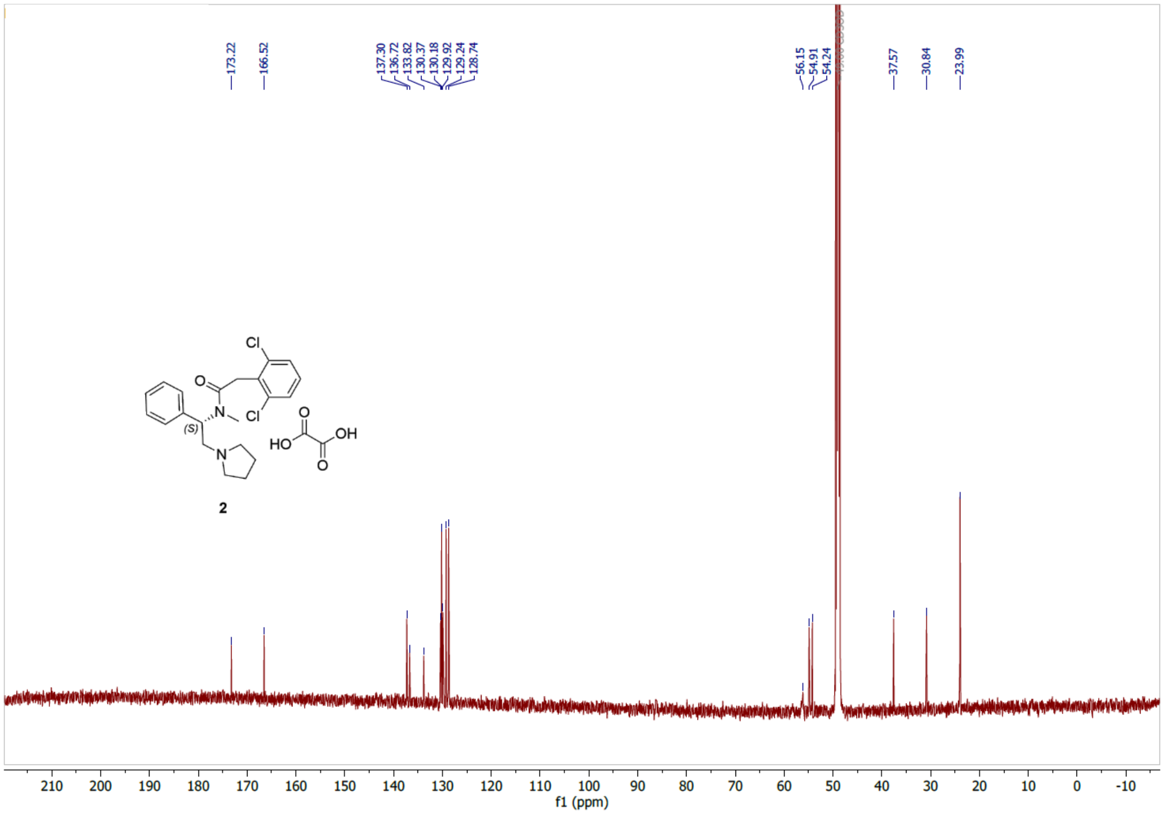
173.22
166.52
137.30
136.72
133.82
130.37
130.18
129.92
129.24
128.74
56.15
54.91
54.24
37.57
30.84
23.99
f1 (ppm)
2

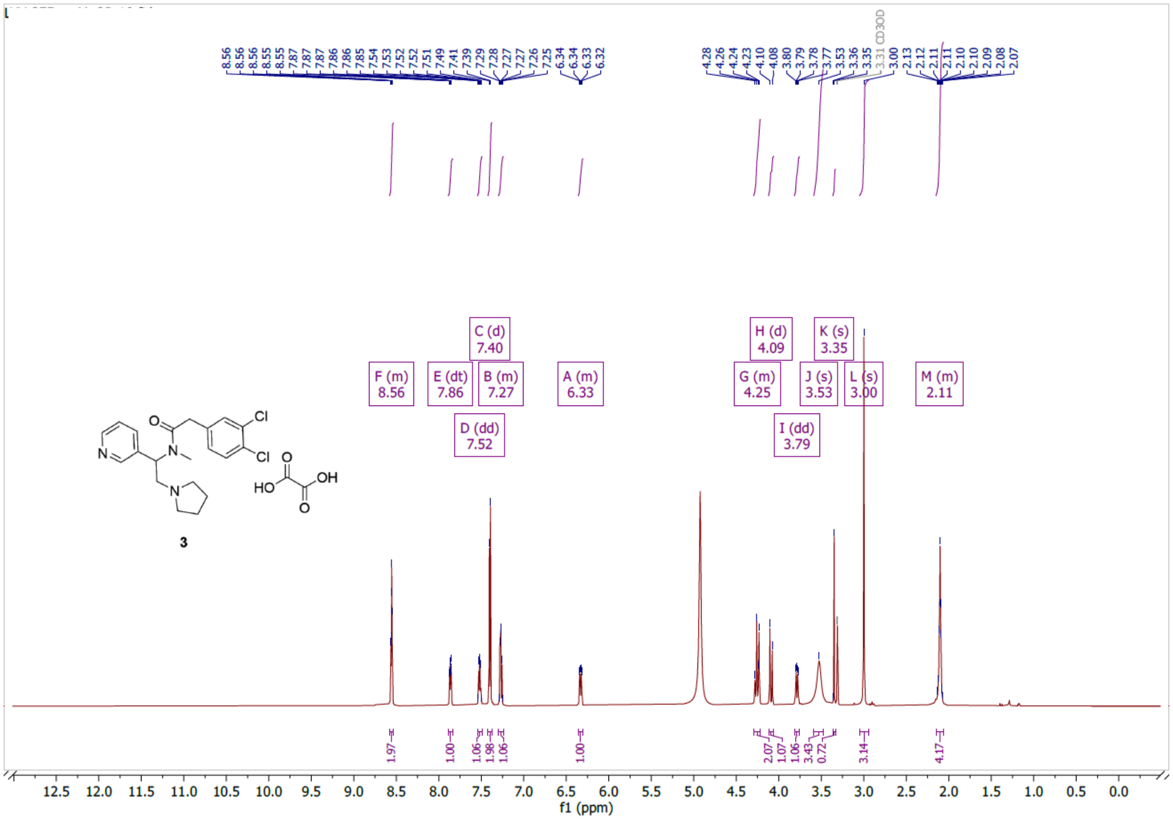
F (m) 8.56
E (dt) 7.86
C (d) 7.40
D (dd) 7.52
B (m) 7.27
A (m) 6.33
H (d) 4.09
G (m) 4.25
I (dd) 3.79
J (s) 3.53
K (s) 3.35
L (s) 3.00
M (m) 2.11
1.97
1.00
1.06
1.98
1.06
1.00
2.07
1.07
1.06
3.43
0.72
3.14
4.17
3.31 CD3OD
Cl
Cl
N
O
N
N
HO
OH
O
O
3
12.5 12.0 11.5 11.0 10.5 10.0 9.5 9.0 8.5 8.0 7.5 7.0 6.5 6.0 5.5 5.0 4.5 4.0 3.5 3.0 2.5 2.0 1.5 1.0 0.5 0.0
f1 (ppm)

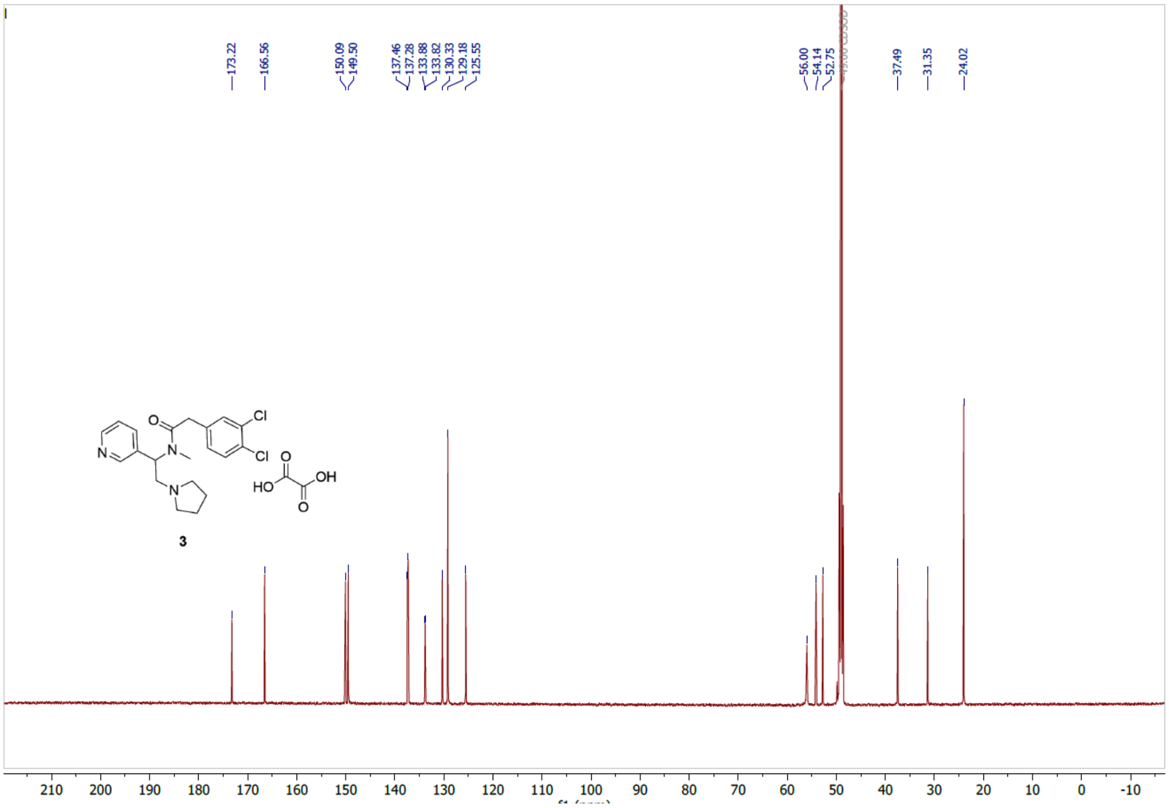
173.22
166.56
150.09
149.50
137.46
137.28
133.88
133.82
130.33
129.18
125.55
56.00
54.14
52.75
49.00 CD3OD
37.49
31.35
24.02
Cl
Cl
N
O
N
N
HO
OH
O
O
3
210 200 190 180 170 160 150 140 130 120 110 100 90 80 70 60 50 40 30 20 10 0 -10

2D HSQC

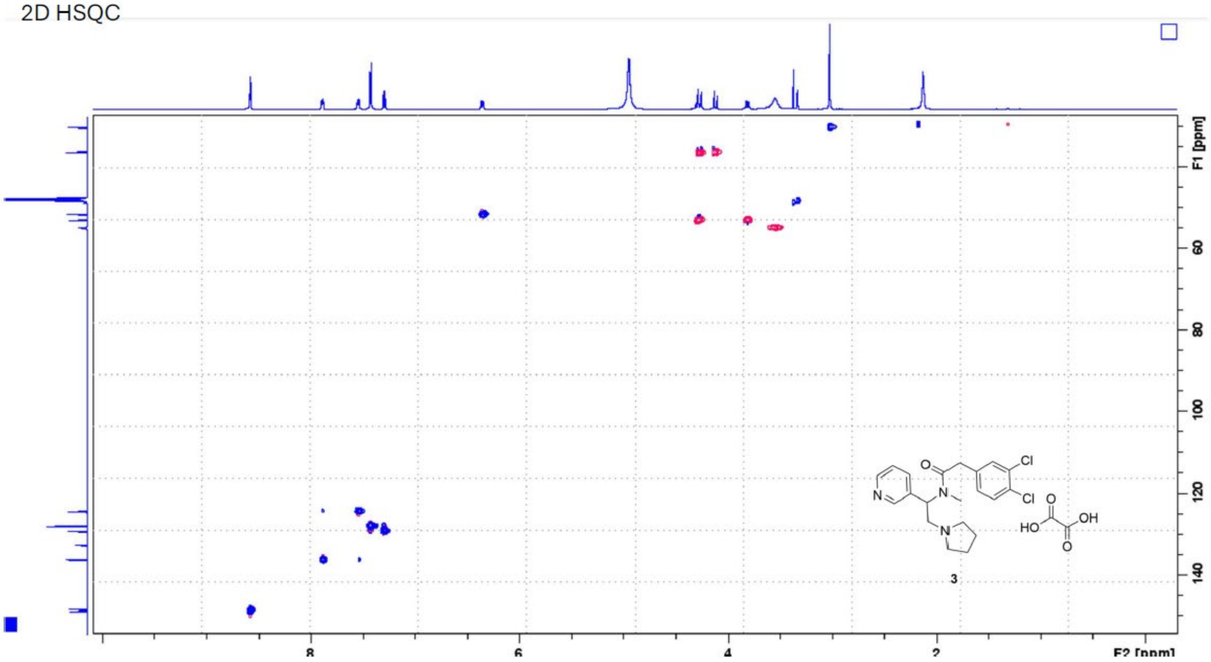

2D HMBC

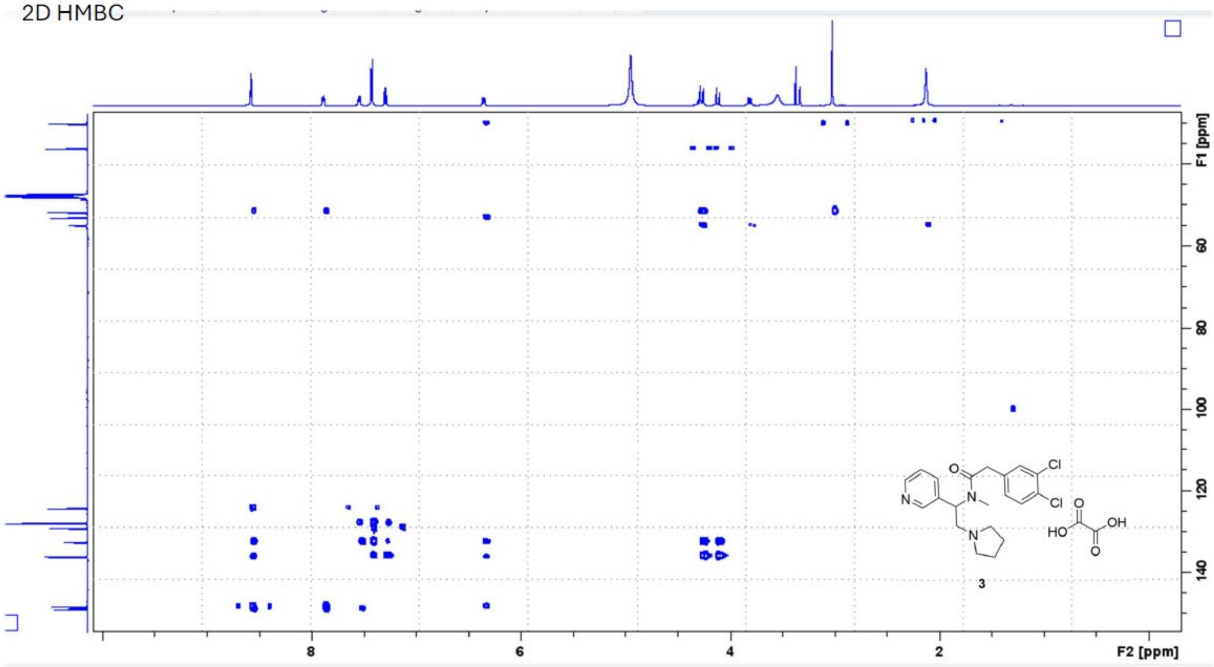

3. Table S1. Summary of statistical analyses

Panel	Description	Statistical analysis	Test statistic
**Figure 2**			
a	Tail withdrawal $logED_{50}$	One-way ANOVA	$F(3, 33) = 10.27$, $p < 0.0001$
a	Tail withdrawal E_{max}	One-way ANOVA	n.s.
b	Tail withdrawal duration	Two-way ANOVA	Interaction: $F(40, 310) = 1.773$, $p < 0.0041$
c	Tail withdrawal duration (KOR KO)	Two-way ANOVA	Interaction: n.s.<br>Treatment: n.s.<br>Time: n.s.
**Figure 3**			
a	Mechanical allodynia Induction phase	Two-way ANOVA	Interaction: $F(8, 400) = 15.97$, $p < 0.0001$
a	Mechanical allodynia Treatment phase	Two-way ANOVA	Interaction: n.s.<br>Treatment: $F(5, 49) = 44.75$, $p < 0.0001$<br>Day: n.s.
b	Thermal allodynia Induction phase	Two-way ANOVA	Interaction: $F(8, 438) = 5.57$, $p < 0.0001$
b	Thermal allodynia Treatment phase	Two-way ANOVA	Interaction: n.s.<br>Treatment: $F(5, 49) = 72.48$, $p < 0.0001$<br>Day: n.s.
**Figure 4**			
a	Openfield 1	Two-way ANOVA	Interaction: $F(44, 275) = 2.76$, $p < 0.0001$
b	Openfield KOR KO	Two-way ANOVA	Interaction: n.s.<br>Treatment: $F(4, 25) = 4.04$, $p = 0.0116$<br>Time: $F(5.78, 145.5) = 11.06$, $p < 0.0001$
c	Openfield 2	Two-way ANOVA	Interaction: n.s.<br>Treatment: $p = 0.0661$<br>Time: $F(6.95, 166.8) = 8.11$, $p < 0.0001$
d	Openfield β-Arrestin2 KO	Two-way ANOVA	Interaction: $F(33, 176) = 2.28$, $p = 0.0003$
e	Openfield 3	Two-way ANOVA	Interaction: $F(33, 429) = 7.29$, $p < 0.0001$
f	Openfield # center	One-way ANOVA	$F(3, 39) = 14.76$, $p < 0.0001$
g	Openfield % center	One-way ANOVA	n.s.
**Figure 5**			
a	Passive wire hang 1	Two-way ANOVA	Interaction: $F(8, 62) = 4.31$, $p = 0.0004$
b	Passive wire hang 2	Two-way ANOVA	Interaction: $F(6, 64) = 9.62$, $p < 0.0001$
c	Rotarod	Two-way ANOVA	Interaction: $F(18, 192) = 15.74$, $p < 0.0001$
d	Rotarod β-Arrestin2 KO	Two-way ANOVA	Interaction: $F(18, 108) = 13.74$, $p < 0.0001$
**Figure 6**			
a	EZM distance	One-way ANOVA	$F(3, 39) = 14.41$, $p < 0.0001$
b	EZM open #	One-way ANOVA	$F(3, 39) = 3.41$, $p < 0.0268$
c	EZM open %	One-way ANOVA	n.s.
d	CPA	Two-way ANOVA	Interaction: n.s.<br>Treatment: n.s.<br>Time: n.s.
**Figure 7**			
a	Frequency	Two-way ANOVA	Interaction: n.s.

			Treatment: n.s.<br>Time: $F(2.9, 14.5) = 9.03$, $p = 0.0014$
b	Frequency KOR KO	Two-way ANOVA	Interaction: n.s.<br>Treatment: n.s.<br>Time: n.s.
c	Tidal vol.	Two-way ANOVA	Interaction: n.s.<br>Treatment: n.s.<br>Time: n.s.
d	Tidal vol. KOR KO	Two-way ANOVA	Interaction: n.s.<br>Treatment: n.s.<br>Time: n.s.

n.s.: not significant.
